# Outbreak of *Cryptosporidium hominis* in northern Sweden: persisting symptoms in a 5-year follow-up

**DOI:** 10.1007/s00436-022-07524-5

**Published:** 2022-04-22

**Authors:** Malin Sjöström, M. Arvidsson, L. Söderström, M. Lilja, J. Lindh, M. Widerström

**Affiliations:** 1 Department of Public Health and Clinical Medicine, Umeå University, Umeå, Sweden; 2 Department of Clinical Microbiology, Umeå University, Umeå, Sweden; 3 Unit of Research, Education and Development–Östersund Hospital, Östersund, Sweden; 4 Department of Public Health and Clinical Medicine, Unit of Research, Education, and Development, Östersund Hospital, Umeå University, Umeå, Sweden; 5 Department of Cell and Molecular Biology, BMC, Uppsala University, Uppsala, Sweden

**Keywords:** Cryptosporidium, Disease outbreaks, Sequelae, Diarrhoea, Post-infectious symptoms

## Abstract

In 2010–2011, a waterborne outbreak of the parasite, *Cryptosporidium hominis*, affected approximately 27,000 inhabitants in the city of Östersund, Sweden. Previous research suggested that post-infectious symptoms, such as gastrointestinal symptoms and joint pain, could persist for up to 2 years after the initial infection. In this study, we investigated whether the parasite caused post-infectious sequelae for up to 5 years after the outbreak. Prospective cohort study. A randomly selected cohort of individuals residing in Östersund at the time of the outbreak was sent a postal questionnaire in 2011. Responders were sent a follow-up questionnaire in 2016 and completed items on whether they experienced a list of symptoms. We examined whether outbreak cases were more likely than non-cases to report post-infectious symptoms 5 years later. We analysed data using logistic regression and calculated odds ratios with 95% confidence intervals. The analysis included 626 individuals. Among the 262 individuals infected during the outbreak, 56.5% reported symptoms at follow-up. Compared to non-cases, outbreak cases were more likely to report watery diarrhoea, diarrhoea, swollen joints, abdominal pain, bloating, joint discomfort, acid indigestion, alternating bowel habits, joint pain, ocular pain, nausea, and fatigue at the follow-up, after adjusting for age and sex. Our findings suggested that cryptosporidiosis was mainly associated with gastrointestinal- and joint-related post-infectious symptoms for up to 5 years after the infection.

**Supplementary Information:**

The online version contains supplementary material available at 10.1007/s00436-022-07524-5.

## Introduction

*Cryptosporidium* is a protozoan parasite that can infect humans and animals. More than 40 species have been identified (Feng et al. 2018), but *C. hominis* and *C. parvum* cause the majority of infections in humans (Feng et al. 2018; Checkley et al. 2015). *Cryptosporidium* is mainly transmitted through the faecal-oral route, either through oocyst-contaminated water or food or through direct contact with an infected person or animal (Chalmers and Davies 2010).

Cryptosporidiosis occurs worldwide and in all age groups (Chalmers and Davies 2010). Many small waterborne outbreaks have been reported globally, but only a few large outbreaks have been reported (Efstratiou et al. 2017). To date, the largest outbreak occurred in Milwaukee, Wisconsin, in 1993. In that outbreak, 400,000 people were infected through the public water supply (Mac Kenzie et al. 1994). In November 2010, Östersund, a city in northern Sweden, experienced a large outbreak of acute diarrhoea, caused by *C. hominis* IbA10G2, which was transmitted through the public water supply. Approximately 27,000 (45%) of 59,000 inhabitants reported symptoms compatible with cryptosporidiosis (Widerstrom et al. 2014).

The most common symptoms of cryptosporidiosis are watery diarrhoea, nausea, vomiting, fever, and abdominal pain. The symptoms typically last a few days to 2–3 weeks (Chalmers and Davies 2010), but the infection can also be asymptomatic (Checkley et al. 2015). Children, particularly those < 2 years old, often display more severe symptoms than adults (Caccio and Chalmers 2016).

Post-infectious symptoms after cryptosporidiosis have been described in several studies (Hunter et al. 2004; Carter et al. 2019 ; Igloi et al. 2018). Long-term sequelae are common after *C. hominis* infections, including diarrhoea, abdominal pain, nausea, fatigue, and headache (Carter et al. 2020). Among children in developing countries, cryptosporidiosis has been associated with increased mortality (Sow et al. 2016), impaired physical fitness, and impaired cognitive function (Guerrant et al. 1999). Two years after the outbreak in Östersund, individuals that reported symptoms of infection during the outbreak (cases) were more likely than those that did not report symptoms of infection (non-cases) to report gastrointestinal symptoms, fatigue, headache, or joint-related symptoms (Lilja et al. 2018). Although a few small studies have followed young children for up to 9 years after sporadic cryptosporidiosis (Guerrant et al. 1999; Berkman et al. 2002), we lack large studies that followed cryptosporidiosis outbreak cohorts for more than 36 months.

The present study aimed to investigate whether post-infectious symptoms persisted for 5 years after a *Cryptosporidium* outbreak.

## Methods

This prospective cohort study was performed in 2016, 5 years after the outbreak of *C. hominis* IbA10G2 in Östersund, Sweden.

### Study population and data collection

Two months after the outbreak in November 2010, we invited 1524 inhabitants in Östersund, representing all ages, to complete a written questionnaire (outbreak questionnaire), which included questions on demographics, onset and symptoms of cryptosporidiosis, and underlying medical conditions. The sample was selected using a random numbers generator. Among 1044 (69%) respondents, 481 (46.1%) were men and 563 (53.4%) were women (Checkley et al. 2015). The response rate was lowest among young adults (48.8%, age 20–29 years), and highest among older adults (> 87%, age > 60 years). Follow-ups were performed at 6 months and 2 years post-outbreak, and the results were reported in detail elsewhere (Lilja et al. 2018; Rehn et al. 2015).

In mid-March 2016, we sent a 5-year follow-up questionnaire (Supplementary File 1), developed for this study, by post to the respondents of the outbreak questionnaire. We included a pre-paid envelope to return the completed questionnaire. For children < 15 years old, we asked parents or guardians to complete the questionnaire. A reminder was sent after 1 month. The respondents reported experiences in the 3 months prior to completing the questionnaire concerning the following post-infectious symptoms: loss of appetite, weight loss, diarrhoea, watery diarrhoea, bloody diarrhoea, abdominal pain, nausea, vomiting, acid indigestion, bloating, a change in bowel habits, headache, eye pain, fatigue, stiff joints, joint pain, swollen joints, and joint discomfort. A blank area was included for reporting any other symptoms. We scanned the returned questionnaires optically and transformed them into an electronic database.

### Case and non-case definitions

A “case” was defined as a respondent that lived in Östersund in mid-January, 2011, and reported, in the outbreak questionnaire, new episodes of diarrhoea (≥ 3 episodes daily), and/or watery diarrhoea, with an onset between November 2, 2010, and January 30, 2011. A “non-case” was defined as any respondent that did not fulfil these criteria during the outbreak.

### Exclusion criteria

We excluded respondents that, in the outbreak questionnaire, reported a prior diagnosis of inflammatory bowel disease (IBD), irritable bowel syndrome (IBS), or “other long-term bowel issues.”

### Data analyses

The study population was stratified according to age and sex. Age was defined as the age at the time of the outbreak. The mean number of symptoms in each group was examined using the Student’s *t*-test. We examined associations between follow-up symptoms and case status with logistic regressions, adjusted for age and sex. The results are expressed as odds ratios (ORs) with 95% confidence intervals (95% CIs). Furthermore, associations between symptoms and case status were examined in different age groups (0–15 years, 16–40 years, 41–65 years, and > 65 years), with logistic regressions adjusted for sex. Missing values were excluded in the analyses. Analyses were performed with the statistical software, SPSS Statistic 24 (IBM, Armonk, NY, USA).

## Results

### Study population

A total of 675 (69.0%) individuals responded to the 5-year follow-up questionnaire. Compared to responders, non-responders were younger (30.8 vs. 46.0 years, *p* < 0.001), and more often men (51.5% vs. 43.7%, *p* = 0.014). There were no differences concerning case status.

**Fig. 1:**
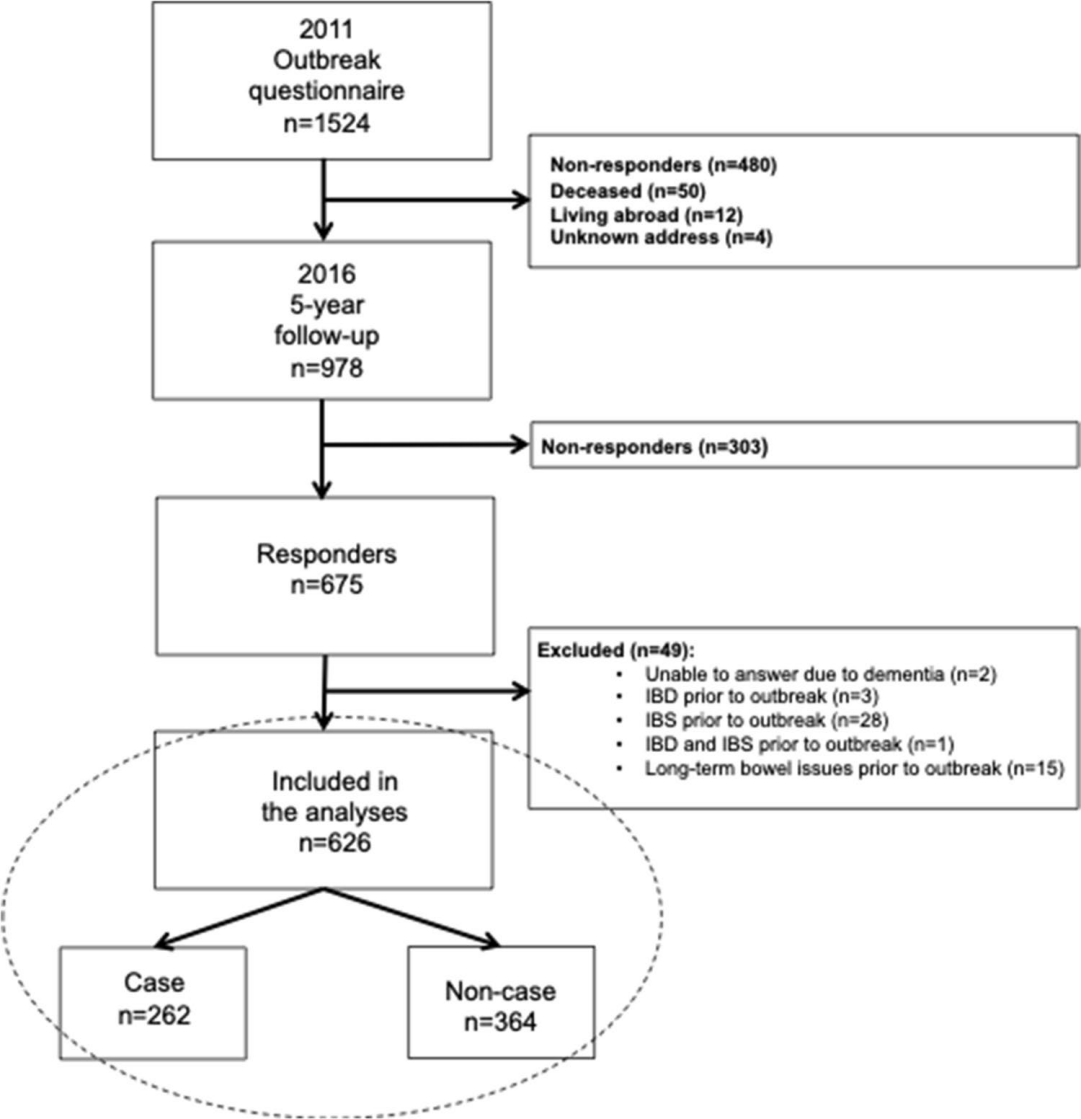
Flow chart of the case selection process. Case = new episodes of diarrhoea (≥ 3 episodes daily), and/or watery diarrhoea between November 1, 2012, and January 31, 2011, in respondent residing in Östersund in mid-January 2011. Non-case = any respondent not fulfilling the case criteria during the outbreak. IBD, inflammatory bowel disease; IBS, irritable bowel syndrome

We excluded 2 individuals unable to answer due to dementia, and 47 individuals that reported IBD, IBS, or other long-term bowel issues prior to the outbreak (Fig. 1).

The final analysis included 626 individuals: 280 (44.7%) men and 346 (55.3%) women. Of these, we defined 262 (41.9%) as cases and 364 (58.1%) as non-cases. The median ages at the time of the outbreak were 43 (range 0–80) years in the case group, and 54 (range 0–92) years in the non-case group (Table 1).

**Table 1:** Demographic characteristics of the study population, grouped by outbreak case status

Characteristic	Case *n* (%)	Non-case *n* (%)	Total *n* (%)
Sex			
Female	145 (55.3)	201 (55.2)	346 (55.3)
Male	117 (44.7)	163 (44.8)	280 (44.7)
Age group, y (at outbreak)			
0–15	44 (16.8)	50 (13.7)	94 (15.0)
16–40	72 (27.5)	68 (18.7)	140 (22.4)
41–65	109 (41.6)	152 (41.8)	261 (41.7)
> 65	37 (14.1)	94 (25.8)	131 (20.9)
Total cases	262	364	626

### Symptoms during follow-up

Five years after the outbreak, 56.5% of the case group and 41.2% of the non-case group reported symptoms during the follow-up period. The case group reported a higher mean number of symptoms (3.8; median = 2, range = 0–17), than the non-case group (2.0; median = 0, range = 0–15; *p* < 0.001). The case group reported that symptoms during the prior 3 months lasted 10 days, compared to 7 days for the non-case group (median). The most frequent symptoms in the case group were headache, fatigue, and bloating.

Compared to the non-case group, the case group was significantly more likely to report watery diarrhoea, diarrhoea, swollen joints, abdominal pain, bloating, joint discomfort, acid indigestion, changes in bowel habits, joint pain, ocular pain, nausea, and fatigue (Table 2). Symptoms associated with case status varied among age groups. Abdominal pain, acid indigestion, and alternating bowel habits were only observed in the > 65-year-old group; diarrhoea, swollen joints, and nausea were only observed in the 41–65-year-old group; and loss of appetite was only observed in the 16–40-year-old group. In the youngest age group (≤ 15 years), watery diarrhoea was the only symptom significantly associated with case status (OR: 11.1, 95% CI: 1.3–92.8).

**Table 2:** Symptoms reported at follow-up by respondents and associations with case status

Symptom	Total			0–15 years	16–40 years	41–65 years	> 65 years
	*N* = 626			*N* = 94	*N* = 140	*N* = 261	*N* = 131
	Cases *n* (%)	Non-cases *n* (%)	OR (95% CI)	OR (95% CI)	OR (95% CI)	OR (95% CI)	OR (95% CI)
Watery diarrhoea	43 (16.8)	14 (4.0)	**4.5 (2.4–8.4)**	**11.1 (1.3–92.8)**	2.5 (0.9–7.0)	**5.0 (2.0–12.5)**	
Diarrhoea	52 (20.2)	23 (6.4)	**3.6 (2.2–6.2)**	3.7 (0.7–19.4)	2.4 (1.0–6.1)	**4.1 (1.9–9.0)**	5.7 (1.0–34.0)
Swollen joints	33 (12.9)	20 (5.8)	**3.0 (1.6–5.4)**		7.5 (0.9–63.4)	**2.7 (1.2–6.2)**	2.7 (0.9–8.1)
Abdominal pain	64 (25.1)	36 (10.1)	**2.6 (1.7–4.1)**	2.0 (0.8–5.0)	2.0 (0.9–4.5)	3.7 (0.7–8.1)	**4.8 (1.1–21.2)**
Bloating	102 (39.4)	73 (20.9)	**2.5 (1.7–3.6)**	2.8 (0.8–8.2)	2.1 (1.0–4.3)	**1.8 (1.1–3.4)**	**5.8 (2.3–14.6)**
Joint discomfort	82 (31.8)	62 (17.7)	**2.4 (1.6–3.6)**	2.7 (0.7–9.6)	**3.5 (1.5–8.4)**	**2.1 (1.2–3.8)**	1.7 (0.7–3.9)
Stiff joints	71 (27.5)	58 (16.7)	**2.3 (1.5–3.4)**	2.2 (0.4–13.0)	**5.8 (1.8–18.2)**	**2.1 (1.2–3.7)**	1.2 (0.5–2.9)
Acid indigestion	62 (23.9)	44 (12.8)	**2.3 (1.5–3.5)**	4.7 (0.9–24.2)	1.9 (0.8–4.6)	1.8 (0.9–3.3)	**3.4 (1.3–8.9)**
Changes in bowel habits	47 (18.1)	32 (9.3)	**2.2 (1.3–3.5)**	2.9 (0.7–11.9)	2.5 (0.9–7.0)	1.5 (0.8–3.0)	**4.3 (1.1–16.2)**
Joint pain	79 (30.4)	65 (18.7)	**2.2 (1.5–3.3)**	1.8 (0.5–7.0)	**4.6 (1.7–12.5)**	**2.5 (1.4–4.4)**	0.8 (0.3–2.0)
Ocular pain	49 (19.3)	33 (9.5)	**2.3 (1.4–3.7)**	2.4 (0.7–7.9)	2.3 (0.9–6.1)	2.1 (0.9–4.6)	2.8 (1.0–7.6)
Nausea	55 (22.0)	35 (9.9)	**2.2 (1.4–3.6)**	1.3 (0.5–3.3)	1.7 (0.7–3.9)	**4.0 (1.8–9.1)**	4.0 (0.8–19.1)
Fatigue	106 (41.4)	85 (24.3)	**2.0 (1.4–2.9)**	1.8 (0.7–4.3)	**2.1 (1.1–4.2)**	**2.2 (1.2–3.8)**	1.6 (0.6–4.2)
Headache	108 (42.2)	103 (29.3)	1.5 (1.0–2.1)	1.2 (0.5–2.8)	1.9 (0.9–3.8)	1.5 (0.8–2.6)	1.1 (0.4–3.4)
Loss of appetite	23 (8.9)	14 (4.1)	2.0 (1.0–4.0)	1.5 (0.4–5.2)	**12.1 (1.5–96.7)**	0.9 (0.2–3.3)	2.6 (0.3–19.5)
Vomiting	20 (7.9)	13 (3.7)	1.8 (0.9–3.7)	1.4 (0.4–4.5)	1.8 (0.5–6.5)	2.7 (0.6–11.5)	
Weight loss	14 (5.5)	10 (2.9)	1.8 (0.8–4.1)	1.4 (0.3–6.8)	1.4 (0.2–8.9)	2.9 (0.5–16.4)	2.5 (0.5–13.3)

Bolded values indicate a significant association

## Discussion

In this prospective cohort study, we demonstrated that post-infectious symptoms persisted for 5 years after a large waterborne outbreak caused by *C. hominis* in northern Sweden. Compared to the non-case group, the case group was more likely to report gastrointestinal symptoms, joint-related symptoms, ocular pain, and fatigue. Middle-aged individuals (41–65 years) seemed to be most affected, particularly by diarrhoea and different joint-related symptoms. The symptoms persisted to a lesser extent in children (≤ 15 years old) than in other age groups.

This study had several strengths. It was conducted by an experienced research group with broad knowledge of infectious medicine and family medicine, and all data analyses were conducted in close collaboration with an experienced statistician. Also, the design with a randomly selected cohort created immediately after the outbreak and prospectively followed for as long as 5 years is unique. Moreover, the overall response rate to the questionnaire was high (69%), and although non-responders were younger and more often men, there was no difference regarding case status at the outbreak among the responders.

There were also limitations to this study. One limitation was that our case definition was based on self-reported symptoms, and we lacked laboratory confirmation. This limitation might have led to misclassification of case status in some individuals. However, during the outbreak in 2010, cryptosporidium was detected in the drinking water; in addition, 149 stool samples collected among individuals with diarrhoea and positive for *C. hominis* were all negative for other gastrointestinal pathogens (Widerstrom et al. 2014). Another limitation was that we did not determine whether participants had a chronic *Cryptosporidium* infection, which could potentially have caused the follow-up symptoms. However, during the 2-year follow-up, study participants were invited to submit stool samples (*n* = 183), and these were all negative for *Cryptosporidium*, based on a standard concentration determination technique with modified Ziehl–Neelsen staining (Lilja et al. 2018). Moreover, it is possible that individuals with chronic intermittent diarrhoea might have been misclassified as cases during the outbreak. These individuals might be more likely to have had similar symptoms at follow-ups, which could have led to an overestimation of the associations. However, we attempted to minimize this effect by excluding individuals that reported a pre-existing IBD or IBS diagnosis, or any other long-term gastrointestinal problems, prior to the outbreak. On the other hand, some participants might have had subclinical *Cryptosporidium* infections during the outbreak (Checkley et al. 2015). If these participants had experienced and reported post-infectious symptoms, they would have contributed to the prevalence of symptoms in the non-case group. Also, as in all observational studies, there is a risk that unknown factors, or factors not measured in the study, affect the risk for both outbreak disease and long-term sequalae. Lastly, we do not know if it were the same persons that experienced the same symptoms over time, and there was also a risk that individuals that were infected during the outbreak (i.e., cases) might be more prone to note, remember, and report on their symptoms, compared to individuals that were not infected.

To our knowledge, no other large studies have conducted such a long follow-up after an acute cryptosporidiosis. Our research group previously reported similar data from the same cohort, where follow-ups after 6–11 months (Rehn et al. 2015) and 2 years (Lilja et al. 2018) demonstrated that the case group was more likely to have gastrointestinal and joint-related symptoms, compared to the non-case group. This can be compared to another Swedish study, 271 individuals with sporadic infections from different types of cryptosporidiosis were followed in 2006–2008. After 25 to 36 months, 15% reported intermittent diarrhoea and 9% reported abdominal pains (Insulander et al. 2013).

Acute gastroenteritis is known to increase the risk of IBS (Litleskare et al. 2018, Thabane et al. 2007). Follow-up studies on *Giardia*, another protozoan parasite that causes gastroenteritis, have shown similar post-infectious symptoms that persisted for up to 10 years (Litleskare et al. 2018). Several putative factors have been implicated in the pathogenesis of IBS, including dysfunction of the innate immune system or the enteric nervous system and alterations in the faecal microbiota (Holtmann et al. 2016), but additional studies are needed to reach a plausible hypothesis for the pathophysiologic mechanism of long-term symptoms after a *Cryptosporidium* infection. However, although the long-term gastrointestinal symptoms reported by many participants in our study were likely to be due to IBS, we could not fully diagnose IBS. A validated questionnaire on the Rome IV criteria is typically used to diagnose IBS (Palsson et al. 2016), but the questionnaire is lengthy, and we were concerned that including the full questionnaire might reduce the overall response rate. Therefore, we decided not to include it in full, but to base our questions concerning gastrointestinal symptoms on those criteria.

Overall, the symptoms most frequently reported in our case group were headache, fatigue, and bloating. In the most affected age group (41–65-year-olds), diarrhoea, joint-related symptoms, nausea, and fatigue were most highly associated with cryptosporidiosis. These data were consistent with results reported in a systematic review based on pooled estimates from 8 studies on the health sequelae of cryptosporidiosis, with follow-up periods of 2–36 months. In that study, the most common long-term sequelae were diarrhoea (25%), abdominal pain (25%), nausea (24%), fatigue (25%), and headache (21%) (Carter et al. 2020).

The group of children (0–15 years old) in our study was small (*n* = 94), and this group reported watery diarrhoea, but no other persisting symptoms. Previous studies have reported that particularly young children (< 2 years old) were vulnerable to acute infections and the consequences (Caccio and Chalmers 2016), and IBS or IBS-like symptoms after cryptosporidiosis occurred at a higher rate in children than in adults (Carter et al. 2019). In contrast, in our cohort, at the 2-year follow-up, the children did not report any significant persistent symptoms, other than headaches (Lilja et al. 2018). However, it is difficult to identify and define sequelae in young children, and adults have been over-represented in most large studies (Carter et al. 2020).

Future directions include continuing to follow the cohort to evaluate whether the post-infectious symptoms will persist for a longer time than 5 years. It is also important to evaluate whether it is the same individuals that experience the symptoms over time. Other possible long-term sequalae would be interesting to explore, such as the suggested association between cryptosporidiosis and colorectal cancer (Sawant et al. 2020). Moreover, it would be interesting to investigate the long-term health economic consequences of the outbreak. There is also a need for more research with a particular focus on long-term persistent symptoms in children. In the meanwhile, clinicians should consider long-term consequences of cryptosporidiosis a possible cause of unexplained gastrointestinal or joint-related symptoms in individuals who have had the infection.

## Conclusion

In summary, our findings indicated that post-infectious symptoms, due to cryptosporidiosis, could persist for up to 5 years, a longer time than previously documented. This finding suggested that the long-term health consequences of cryptosporidiosis may be underestimated, both on an individual level and on the global level.

## Supplementary Information

Below is the link to the electronic supplementary material.

Supplementary file1 (PDF 112 KB)

## Data Availability

The datasets used in the current study are available from the corresponding author on reasonable request.

## References

[CR1] Berkman DS, Lescano AG, Gilman RH, Lopez SL, Black MM (2002). Effects of stunting, diarrhoeal disease, and parasitic infection during infancy on cognition in late childhood: a follow-up study. Lancet.

[CR2] Caccio SM, Chalmers RM (2016). Human cryptosporidiosis in Europe. Clin Microbiol Infect.

[CR3] Carter BL, Stiff RE, Elwin K, Hutchings HA, Mason BW, Davies AP (2019). Health sequelae of human cryptosporidiosis—a 12-month prospective follow-up study. Eur J Clin Microbiol Infect Dis.

[CR4] Carter B, Chalmers R, Davies A (2020). Health sequelae of human cryptosporidiosis in industrialised countries: a systematic review. Parasit Vectors.

[CR5] Chalmers RM, Davies AP (2010). Minireview: clinical cryptosporidiosis. Exp Parasitol.

[CR6] Checkley W, White AC Jr, Jaganath D, Arrowood MJ, Chalmers RM, Chen XM et al (2015) A review of the global burden, novel diagnostics, therapeutics, and vaccine targets for cryptosporidium. Lancet Infect Dis 15:85–94. 10.1016/S1473-3099(14)70772-8 10.1016/S1473-3099(14)70772-8 PMC4401121 25278220

[CR7] Efstratiou A, Ongerth JE, Karanis P (2017). Waterborne transmission of protozoan parasites: review of worldwide outbreaks—an update 2011–2016. Water Res.

[CR8] Feng Y, Ryan UM, Xiao L (2018). Genetic diversity and population structure of cryptosporidium. Trends Parasitol.

[CR9] Guerrant DI, Moore SR, Lima AAM, Patrick PD, Schorling JB, Guerrant RL (1999). Association of early childhood diarrhea and cryptosporidiosis with impaired physical fitness and cognitive function four-seven years later in a poor urban community in northeast Brazil. Am J Trop Med Hyg.

[CR10] Holtmann GJ, Ford AC, Talley NJ (2016) Pathophysiology of irritable bowel syndrome. Lancet Gastroenterol Hepatol 1:133–146. 10.1016/S2468-1253(16)30023-1 10.1016/S2468-1253(16)30023-1 28404070

[CR11] Hunter PR, Hughes S, Woodhouse S, Raj N, Syed Q, Chalmers RM (2004). Health sequelae of human cryptosporidiosis in immunocompetent patients. Clin Infect Dis.

[CR12] Igloi Z, Mughini-Gras L, Nic Lochlainn L, Barrasa A, Sane J, Mooij S (2018). Long-term sequelae of sporadic cryptosporidiosis: a follow-up study. Eur J Clin Microbiol Infect Dis.

[CR13] Insulander M, Silverlås C, Lebbad M, Karlsson L, Mattsson JG, Svenungsson B (2013). Molecular epidemiology and clinical manifestations of human cryptosporidiosis in Sweden. Epidemiol Infect.

[CR14] Lilja M, Widerstrom M, Lindh J (2018). Persisting post-infection symptoms 2 years after a large waterborne outbreak of *Cryptosporidium hominis* in northern Sweden. BMC Res Notes.

[CR15] Litleskare S, Rortveit G, Eide GE, Hanevik K, Langeland N, Wensaas KA (2018). Prevalence of irritable bowel syndrome and chronic fatigue 10 years after Giardia infection. Clin Gastroenterol Hepatol.

[CR16] Mac Kenzie WR, Hoxie NJ, Proctor ME, Gradus MS, Blair KA, Peterson DE (1994). A massive outbreak in Milwaukee of cryptosporidium infection transmitted through the public water supply. N Engl J Med.

[CR17] Palsson OS, Whitehead WE, van Tilburg MA, Chang L, Chey W, Crowell MD (2016). Rome IV diagnostic questionnaires and tables for investigators and clinicians. Gastroenterology.

[CR18] Rehn M, Wallensten A, Widerstrom M, Lilja M, Grunewald M, Stenmark S (2015). Post-infection symptoms following two large waterborne outbreaks of *Cryptosporidium hominis* in Northern Sweden, 2010–2011. BMC Public Health.

[CR19] Sawant M, Baydoun M, Creusy C, Chabé M, Viscogliosi E, Certad G (2020). *Cryptosporidium* and colon cancer: cause or consequence?. Microorganisms.

[CR20] Sow SO, Muhsen K, Nasrin D, Blackwelder WC, Wu Y, Farag TH (2016). The burden of Cryptosporidium diarrheal disease among children < 24 months of age in moderate/high mortality regions of subSaharan Africa and south Asia, utilizing data from the global enteric multicenter study (GEMS). PLoS Negl Trop Dis.

[CR21] Thabane M, Kottachchi DT, Marshall JK (2007). Systematic review and meta-analysis: the incidence and prognosis of post-infectious irritable bowel syndrome. Aliment Pharmacol Ther.

[CR22] Widerstrom M, Schonning C, Lilja M, Lebbad M, Ljung T, Allestam G (2014). Large outbreak of *Cryptosporidium hominis* infection transmitted through the public water supply, Sweden. Emerg Infect Dis.

