# Supplementary material for: Outbreak of Cryptosporidium hominis in northern Sweden: persisting symptoms in a 5-year follow-up
Source: Parasitol Res. 2022 Apr 22;121(7):2043–9. doi: 10.1007/s00436-022-07524-5 (PMC9192462; doi:10.1007/s00436-022-07524-5)
Supplement: Supplementary file 1 — Supplementary file1 (PDF 112 KB) [file 436_2022_7524_MOESM1_ESM.pdf]

Survey follow-up after the Cryptosporidium outbreak winter 2010/2011. (Adults)
(Tick the boxes using a ballpoint pen)

Age: ___ (years) **I am:** male female

1a. During the last three months, have you had any of the following symptoms? Yes No

- diarrhoea with 3 or more loose stools per day
- watery diarrhoea
- bloody diarrhoea
- abdominal pain / abdominal cramps
- vomiting
- nausea
- fever, more than 38 degrees
- headache
- joint discomfort
- eye pain
- fatigue

(If you have answered no to all the above, please proceed to question 2a)

1b. For how many days during the last three months did you experience any of the symptoms in question 1a? ____ (number of days with symptoms)

1c. Do you believe any of the symptoms in question 1a started after the outbreak of cryptosporidium?
Yes, some of the symptoms started then
No, the symptoms did not start then

2a. During the last three months, have you had any of the following? Yes No

- constipation
- altering bowel habits
- bloating
- acid indigestion
- loss of appetite
- weight loss

2b. If you have had any of the symptoms in question 2a, for how many days during the last three months did you experience the symptoms? ___ (number of days with symptoms)

3a. During the last three months, have you had any of the following? Yes No

- stiff joints
- joint pain
- swollen joints

3b. If you have had any of the symptoms in question 3a, for how many days during the last three months did you experience the symptoms? ___ (number of days with symptoms)

4. During the <u>last three months</u>, have you been home from work or studies because of any of the symptoms in question 2a and/or 3a? Yes No

If yes, for how long in total? _____ number of days

5. During the <u>last three months</u>, have you visited a health care center for any of the symptoms in question 1a, 2a and/or 3a? (more than one answer possible)

No
Yes, primary care center
Yes, hospital

6. Do you have any of the following symptoms or illnesses: Yes No

- stomach ulcer
- irritable bowel syndrome (IBS)
- inflammatory bowel disease (Ulcerative colitis or Crohn's disease)
- gluten intolerance
- lactose intolerance
- other long-term bowel issues
- diabetes
- COPD / asthma
- heart failure
- rheumatic joint disease
- cancer

7. Are you currently treated with any of the following medicines? Yes No

- medicine for gastric ulcer / acid rejections
- (e*.g. Omeprazol, Losec, Nexium)*
- cortisone
- cell cures or other drugs that reduce the immune system?

8. Do you think you have any current symptoms caused by the Cryptosporidium infection 2010-2011? Yes No

If yes, please leave any comments or information on the back of this paper

9. Do you feel worried about your health?

Put a cross in the box that best suits you. 0 = not at all worried and 10 = very worried.

10. Did you have infection with Cryptosporidium in 2010-2011? Yes No

11. What is your occupation today? Yes No
(more than one answer possible)

- Full time work or studies
- Part time work or studies
- Full time sick leave since >1 month
- Part time sick leave since >1 month
- Retired or disability pension
- Other: _________

Thank you for answering the questions!
Please return the completed questionnaire as soon as possible using the enclosed envelope.

Please leave any additional comments or information below or on a separate paper.
